# Structure-Activity Relationship of Mono-Ion Complexes for Plasmid DNA Delivery by Muscular Injection

**DOI:** 10.3390/pharmaceutics13010078

**Published:** 2021-01-08

**Authors:** Amika Mori, Yuki Kobayashi, Kei Nirasawa, Yoichi Negishi, Shoichiro Asayama

**Affiliations:** 1 Department of Applied Chemistry, Tokyo Metropolitan University, Hachioji, Tokyo 192-0397, Japan; mori-amika@ed.tmu.ac.jp (A.M.); kobayashi-yuki4@ed.tmu.ac.jp (Y.K.); 2 Department of Drug Delivery and Molecular Biopharmaceutics, Tokyo University of Pharmacy and Life Sciences, Hachioji, Tokyo 192-0392, Japan; y131192@toyaku.ac.jp (K.N.); negishi@toyaku.ac.jp (Y.N.)

**Keywords:** mono-ion complex, monocationic poly(ethylene glycol), plasmid DNA delivery, muscular injection, amide bond spacer, ester bond spacer

## Abstract

The structure-activity relationship of mono-ion complexes (MICs) for plasmid DNA (pDNA) delivery by muscular injection is demonstrated. MICs were formed between pDNA and monocationic poly(ethylene glycol) (PEG) macromolecules. As monocationic PEGs, the ω-amide-pentylimidazolium (APe-Im) end-modified PEGs with a stable amide (Am) and hydrolytic ester (Es) bond, that is, APe-Im-Am-PEG and APe-Im-Es-PEG, respectively, are synthesized. The difference between the APe-Im-Am-PEG and APe-Im-Es-PEG was only a spacer structure between a terminal cation and a PEG chain. The resulting pDNA MICs with APe-Im-Am-PEG at a charge ratio (+/−) of 32 or 64 were more stable than those with APe-Im-Es-PEG in the presence of serum proteins. The highest gene expression by muscular injection was achieved using the APe-Im-Am-PEG/pDNA MIC at a charge ratio (+/−) of 32 with a smaller particle diameter of approximately 50 nm, as compared to that charge ratio of 64. Consequently, the pDNA MIC with the monocationic PEG with a stable amide spacer, as compared to a hydrolytic ester spacer, is considered to be suitable for the highest gene expression by muscular injection.

## 1. Introduction

Recently, in 2019, plasmid DNA (pDNA) encoding the human hepatocyte growth factor (HGF) gene was first approved for peripheral arterial disease in Japan. From the first human clinical trial of HGF gene therapy, the approval took approximately 15 years. The intramuscular injection of naked pDNA encoding the HGF gene was tolerated [1,2]. pDNAs have advantageously produced many therapeutic RNAs by transcription and many therapeutic proteins by translation [3,4,5]. Clinical application generally desires that the gene expression from pDNA is preserved in vivo for sustainable production of therapeutic RNAs and proteins [6,7,8]. However, since pDNA is a polyanion with a high molecular weight of approximately 10^6^, pDNA has many barriers to the application of effective medicine. An effective pDNA delivery system is therefore necessary to realize clinical therapy.

The pDNA delivery system widely used the various polyion complexes (PICs) between pDNA and polycations by electrostatic interaction for pDNA transfection in vitro [9,10], as well as our previous PICs [11,12,13,14,15,16,17], and in vivo [18,19]. In Asayama’s recent review [20], various molecular designs of polymer-based carriers for pDNA delivery in vitro and in vivo have been reported. The pDNA PICs with polycations usually have positive-charged surfaces, which often result in fast elimination of the pDNA PICs from blood circulation and off-target delivery of pDNAs. Furthermore, the positive-charged surfaces cause aggregation with serum albumin, coagulation of red blood cells, and cytotoxicity. These disadvantages are considered to be the reason why polycations are difficult to use for pDNA delivery in vivo [21].

From a more anatomical point of view, especially, the tumor stromal barrier is one of the critical factors that make it difficult to translate the pDNA expression efficiency from in vitro to in vivo, including pDNA muscular injection in our recent study [22], because intratumoral extravasation and penetration of pDNA PICs are very limited [23,24]. The most common strategy to improve these undesirable properties is shielding with nonionic hydrophilic polymers such as poly(ethylene glycol) (PEG), called PEGylation [25]. The PEGylation prolongs blood circulation time [26] and mediates permeation diffusively in tumor stroma by minimizing electrostatic interactions with an extracellular matrix [27].

To cross the barrier of tumor stroma by the diffusion-mediated PIC permeation, our original strategy of the mono-ion complex (MIC) formation between pDNA and the mono-cationic PEG has been established [28,29]. As the mono-cationic PEG, the alkylimidazolium end-modified PEG (R-Im-PEG) has been designed for the tuning of the electrostatic interaction with pDNA by the length of the alkyl chain [28]. Based on the molecular design of the R-Im-PEG, the pDNA MIC formation with the R-Im-PEG has been achieved, resulting in the suppression of the increase in the particle size by the avoidance of the multivalent electrostatic interaction of the PIC formation. The resulting smaller particle size is considered to be suitable for the diffusion-mediated permeation by the PEGylation. Furthermore, to stabilize the pDNA MIC with the R-Im-PEG by hydrogen bond formation, we synthesized ω-amide-pentylimidazolium end-modified PEG (APe-Im-PEG) by mimicking an asparagine residue of a DNA binding protein to form the hydrogen bond with pDNA via a base of adenine [29].

Including our MIC, although PEGylation enhances biocompatibility to suppress nonspecific interaction with mainly serum proteins, PEGylation often blocks the accessibility to target cells [30,31]. To access target cells in spite of pDNA protection by PEGylation, we have already synthesized APe-Im-PEG with an ester bond [32], instead of an amide bond in our previous MICs [28,29], to be gradually hydrolyzed for cleavage of PEG, that is, APe-Im-E-PEG, resulting in sustainable gene expression under optimal conditions. Notably, the morphology of the resulting pDNA MIC seems to be not spherical and less condensed, suggesting flexible structure [32]. The in vivo transfection activity mediated by our pDNA/PEG MIC is higher than that mediated by naked pDNA to exhibit no in vitro significant protein expression. The above background led us to examine whether the ester bond is superior to the amide bond for muscular injection by our previous MIC.

In this study, the structure-activity relationship of MICs for pDNA delivery by muscular injection is demonstrated. As monocationic PEGs, the APe-Im-PEGs with a stable amide (Am) and hydrolytic ester (Es) bond, that is, APe-Im-Am-PEG and APe-Im-Es-PEG, respectively, were synthesized. The difference between the APe-Im-Am-PEG and APe-Im-Es-PEG was only a spacer structure between a terminal cation and a PEG chain, so that we could easily focus on the importance of the spacer structure, Am or Es, for the MIC activity. The properties of the resulting MICs, such as particle size, morphology, stability in serum protein, cell viability, and pDNA expression in skeletal muscle were compared between the APe-Im-Am-PEG and APe-Im-Es-PEG.

## 2. Materials and Methods

### 2.1. Materials

α-Aminoethyl-ω-methoxy, polyoxyethylene (aminoethyl-PEG) (Mw 2000) was purchased from NOF corporation (Tokyo, Japan). Poly(ethylene glycol) methyl ether (hydroxyethyl-PEG) (average M_n_ ~ 2000) was purchased from Sigma-Aldrich (St. Louis, MO, USA). 1-Imidazoleacetic acid was purchased from Tokyo Chemical Industry Co., LTD. (Tokyo, Japan). 6-Bromohexanamide was purchased from Combi-Blocks Inc. (San Diego, CA, USA). All other chemicals of a special grade were used without further purification.

### 2.2. Synthesis of APe-Im-Am-PEG and APe-Im-Es-PEG

**Figure 1:**
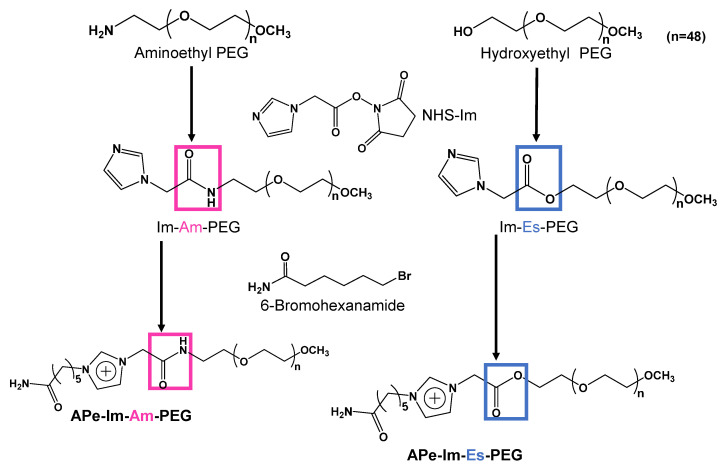
Synthesis scheme of APe-Im-Am-PEG and APe-Im-Es-PEG.

A typical procedure to synthesize APe-Im-Am-PEG is as follows (Figure 1): 1-Imidazoleacetic acid (126.53 mg: 1.0 mmol), *N*-hydroxysuccinimide (NHS) (115.72 mg: 1.0 mmol), and *N*,*N*-dicyclohexylcarbodiimide (DCC) (206.69 mg: 1.0 mmol) were mixed in 10 mL of *N*,*N*-dimethylformamide (DMF) in the presence of 139 μL (1.0 mmol) of triethylamine (TEA), followed by the incubation at 50 °C for 20 h to synthesize an active ester (NHS-Im). After the incubation, aminoethyl-PEG (400.72 mg: 0.2 mmol) was added to the resulting mixture, followed by further incubation at 50 °C for three days. The resulting mixture was dialyzed against distilled water using the Spectra/Por CE membrane (molecular weight cutoff of 100–500), followed by filtration with a 0.22 μm cellulose acetate filter to remove undesirable precipitates. After freeze-drying, the mixture of the resulting sample (Im-Am-PEG) (138.19 mg: 0.063 mmol) and 6-bromohexanamide (244.85 mg: 1.26 mmol) in 5 mL of DMF in the presence of 8.66 μL (0.063 mmol) of TEA was incubated at 50 °C for three days. Then, the dialysis of the resulting mixture against distilled water using the Spectra/Por CE membrane (molecular weight cutoff of 100–500) was carried out, followed by freeze-drying to obtain APe-Im-Am-PEG.

A typical procedure to synthesize APe-Im-Es-PEG, which is almost the same as the above scheme except for the use of hydroxyethyl-PEG, is as follows (Figure 1): 1-Imidazoleacetic acid (126.33 mg: 1.0 mmol), *N*-hydroxysuccinimide (NHS) (115.61 mg: 1.0 mmol), and *N*,*N*-dicyclohexylcarbodiimide (DCC) (207.50 mg: 1.0 mmol) were mixed in 10 mL of *N*,*N*-dimethylformamide (DMF), followed by incubation at 50 °C for 20 h to synthesize the active ester (NHS-Im). After the incubation, hydroxyethyl-PEG (400.42 mg: 0.2 mmol) was added to the resulting mixture, followed by further incubation at 50 °C for three days. The dialysis of the resulting mixture against distilled water with the Spectra/Por CE membrane (molecular weight cutoff of 100–500) was carried out, followed by freeze-drying. The mixture of the resulting sample (Im-Es-PEG) (80.87 mg: 0.038 mmol) and 6-bromohexanamide (147.70 mg: 0.76 mmol) in 5 mL of DMF in the presence of 5.22 μL (0.038 mmol) of TEA was incubated at 50 °C for three days. Then, the dialysis of the resulting mixture against distilled water using the Spectra/Por CE membrane (molecular weight cutoff of 100–500) was carried out, followed by freeze-drying to obtain APe-Im-E-PEG.

### 2.3. ^1^H NMR Spectroscopy

The resulting polymer (5 mg) was dissolved in 600 μL of D_2_O (99.8 atom% deuterium; Acros, NJ, USA). The ^1^H NMR spectra (500 MHz) were measured by a Bruker AV500 spectrometer (Billerica, MA, USA).

### 2.4. Gel Filtration Chromatography

Gel Filtration Chromatography (GFC) was carried out by use of a JASCO PU-980 pumping system (Tokyo, Japan) at the flow rate of 1.0 mL/min with a Shodex OHpak SB-804 HQ column (Showa Denko K. K., Tokyo, Japan). As a mobile phase, phosphate-buffered saline without divalent cations, PBS(−), was used. One hundred microliters of 1 mg/mL samples were injected into the column during the incubation of the samples at 37 °C for seven days in PBS(−). The detection of the eluate was carried out with both a RI detector (RI-1530; Jasco) and a UV detector (UV-2077; Jasco).

### 2.5. Agarose Gel Retardation Assay

A stock solution of the APe-Im-Am-PEG or APe-Im-Es-PEG (1.0–8.6 μL) and the dilution (300 ng of pDNA) of pDNA stock solution with H_2_O were mixed where the final volume was adjusted to 13.5 μL at various [ω-Amide-pentylimidazolium (Cation)]_PEG_/[Phosphate]_pDNA_ ratios, followed by incubation at 37 °C for 24 h. After mixing with a loading buffer (1.5 μL), the resulting sample was loaded onto a 1% agarose gel containing 1 μg/mL ethidium bromide. Gel electrophoresis (50 V, 30 min) was performed at room temperature in a TAE buffer (Tris-acetate, EDTA), followed by the visualization of the pDNA bands under UV irradiation. For the stability assay of the MIC, the electrophoresis was performed after the MICs were incubated at 37 °C for 5 min or 10 min in the presence of 10% fetal bovine serum (FBS).

### 2.6. Particle Size Measurement

A dynamic light scattering (DLS) method by an electrophoresis light scattering spectrophotometer (ELS-Z2, Otsuka Electronics Co., Ltd., Tokyo, Japan) determined the size of the pDNA (5 μg) incubated at 37 °C for 24 h with the APe-Im-Am-PEG or APe-Im-Es-PEG at various [ω-Amide-pentylimidazolium (Cation)]_PEG_/[Phosphate]_pDNA_ ratios in 100 μL of PBS(−). The zeta potential of the resulting sample was measured by ELS with electrodes.

### 2.7. Transmission Electron Microscopy (TEM) Observations of the MIC Structures

The mixture of 2 μL of a twice-diluted MIC solution with 2 μL of 2% uranyl acetate on ice was used for a TEM sample solution to observe the resulting MIC. After a TEM grid (Nisshin EM Co., Tokyo, Japan) was hydrophilized by an Eiko IB-3 ion coater (Eiko Engineering Co., Ltd., Shimane, Japan), the hydrophilized grid was dipped into the sample solution for 40 s. The excess solution was blotted away. The observation of the resulting grids was carried out by a JEM-1400 Bio-TEM (JEOL Ltd., Tokyo, Japan) operated at an acceleration voltage of 120 kV.

### 2.8. Cell Viability Assay

As a representative cell, human hepatoma cell line HepG2 cells (from Riken Bioresource Center Cell Bank) were cultured in tissue culture flasks in Dulbecco’s modified Eagle’s medium supplemented with 10% heat-inactivated FBS. The cells were seeded at 1 × 10^4^ cells/well in a 96-well plate, followed by incubation overnight at 37 °C in a 5% CO_2_ incubator. The cells were treated with the APe-Im-Am-PEG or APe-Im-Es-PEG (0–10 mg/mL), followed by incubation for 24 h at 37 °C. By additional incubation for 4 h, the cell viability was measured by the Alamar Blue assay [33] in triplicate.

### 2.9. In Vivo Gene Delivery to the Skeletal Muscles by MICs

In vivo gene delivery to the skeletal muscles of mice with the APe-Im-Am-PEG or APe-Im-Es-PEG was carried out using previously described methods [34]. Briefly, ICR mice (five weeks old, male) were anesthetized with pentobarbital. The MICs of pDNA (5 μg) with the APe-Im-Am-PEG or APe-Im-Es-PEG at the [ω-Amide-pentylimidazolium (Cation)]_PEG_/[Phosphate]_pDNA_ ratios of 32 and 64 in 35 μL of PBS(−) were incubated at 37 °C for 24 h, followed by injection into the tibialis muscles of the ICR mice. One week or two weeks after the injection, the whole tibialis muscles were collected and homogenized in a lysis buffer (0.1 M Tris-HCl (pH 7.8), 0.1% Triton X-100, and 2 mM EDTA). Luciferase activity was measured with a luminometer (LB96V, Belthold Japan Co. Ltd., Tokyo, Japan) according to a luciferase assay system (Promega, Madison, WI, USA). The luciferase activity is normalized by relative light units (RLU) per mg of protein. The pcDNA3-Luc plasmid was derived from pGL3-basic (Promega, Madison, WI, USA) and used as a pDNA encoding the firefly luciferase gene under the control of a cytomegalovirus promoter.

### 2.10. Animals

The use of animals and relevant experimental procedures were approved by the Tokyo University of Pharmacy and Life Science Committee on the Care and Use of Laboratory Animals. The animal experiment protocol approval number is P20–54.

### 2.11. Statistical Analysis

Statistical analysis was performed using the two-sample equal variance student’s *t*-test.

## 3. Results and Discussion

### 3.1. Synthesis and Hydrolysis Properties of APe-Im-Am-PEG and APe-Im-Es-PEG

Figure 1 shows the synthesis scheme of APe-Im-Am-PEG and APe-Im-Es-PEG to form MICs. First, we reacted aminoethyl-PEG and hydroxyethyl-PEG for the synthesis of Im-Am-PEG and Im-Es-PEG, respectively, with 1-imidazoleacetic acid by condensation reaction using NHS and DCC. The resulting Im-Am-PEG and Im-Es-PEG were subsequently reacted with 6-bromohexaneamide for the quaternization of the imidazole group of PEGs. The ^1^H NMR spectrum indicated the characteristic signal of the methylene protons neighboring an amide (Am) or ester (Es) group as well as a *ω*-amide-pentyl group (APe), an imidazolium group (Im), and a PEG (PEG) (see Figure S1 in the Supplementary Materials). From the suitable signal ratio, we confirmed the successful synthesis of the *ω*-amide-pentylimidazolium end-modified PEGs with an amide (Am) and ester (Es) bond, that is, APe-Im-Am-PEG and APe-Im-Es-PEG, respectively.

To examine the hydrolysis properties of the resulting APe-Im-Am-PEG and APe-Im-Es-PEG, we carried out a GFC experiment under physiological conditions (see Figure S2 in the Supplementary Materials). During the incubation of APe-Im-Es-PEG for seven days, the retention time (RT) of imidazolium detected by absorbance (ABS) at 300 nm was gradually delayed. Namely, the later peak from the dissociated imidazolium group (RT 9.8 min) gradually increased against the earlier peak from the intact APe-Im-Es-PEG (RT 9.0 min) (Figure S2B). Conversely, in the case of APe-Im-Am-PEG, the later peak from the dissociated imidazolium group (RT 9.8 min) was almost constant against the earlier peak from the intact APe-Im-Am-PEG (RT 8.9 min) (Figure S2A). These results suggest that the APe-Im-Es-PEG is considered to be gradually hydrolyzed under physiological conditions with APe-Im-Am-PEG being stable.

### 3.2. Formation of the pDNA MIC with APe-Im-Am-PEG and APe-Im-Es-PEG

**Figure 2:**
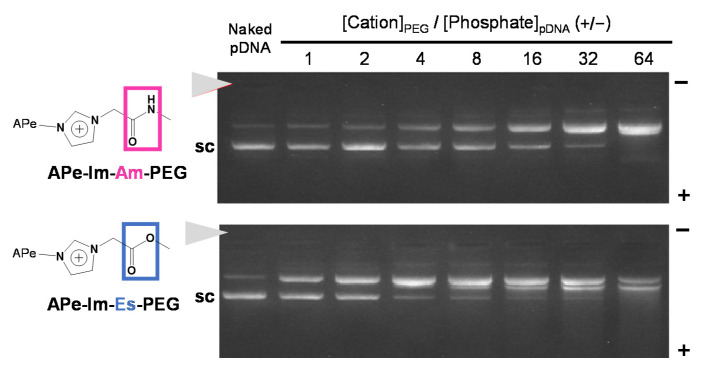
The pDNA MIC formation with APe-Im-Am-PEG or APe-Im-Es-PEG assessed by agarose gel electrophoresis. The mixing charge ratios of the *ω*-amide-pentylimidazolium group of APe-Im-Am-PEG or APe-Im-Es-PEG to phosphate group of pDNA ([*ω*-amide-pentylimidazolium (Cation)]_PEG_/[Phosphate]_pDNA_) (+/−) are indicated. The solid arrowhead indicates the well where each sample was loaded. The migration band of super-coiled pDNA is indicated by “sc”.

To compare the pDNA MIC formation ability of the resulting APe-Im-Am-PEG and APe-Im-Es-PEG, as shown in Figure 2, we carried out agarose gel electrophoresis after one-day incubation with pDNA because of the monovalent electrostatic interaction to form the MIC, although most PIC formation by multivalent electrostatic interaction is examined after a shorter time (approximately 1 h) incubation [35]. In the presence of the APe-Im-Am-PEG or APe-Im-Es-PEG, the migration of a super-coiled pDNA band (“sc” in Figure 2) was almost completely retarded above the [*ω*-Amide-pentylimidazolium (Cation)]_PEG_/[Phosphate]_pDNA_ ratio of 32, suggesting the pDNA MIC formation with the APe-Im-Am-PEG or APe-Im-Es-PEG. Although the pDNA MIC formation ability of APe-Im-Es-PEG seems to be a little higher than that of APe-Im-Am-PEG, because of the lower intensity of the remaining super-coiled pDNA band (“sc” in Figure 2) at the [Cation]_PEG_/[Phosphate]_pDNA_ ratio of 4 in case of APe-Im-Es-PEG, it is difficult to conclude whether the most suitable ability to form the pDNA MIC is with the APe-Im-Am-PEG or with APe-Im-Es-PEG at present. The retardation properties, which did not exist at the loading site (solid arrowhead), are considered to be characteristic of the MIC with negative zeta potential (Table 1). The resulting negative zeta potential is considered to be stable in the anionic serum-containing environment because the zeta potential values of the pDNA PICs with poly(L-lysine) (PLL: polycation)-PEG block copolymers are approximately 2–6 mV to work in anionic serum proteins [36]. Especially, the APe-Im-Am-PEG/pDNA and APe-Im-Es-PEG MICs mixed at the charge ratio ([*ω*-Amide-pentyl- imidazolium (Cation)]_PEG_/[Phosphate]_pDNA_) (+/−) of 32 have a small particle size below 50 nm (Table 1), as compared to the above PLL-PEG/pDNA PICs with an approximately 90–100 nm particle size [36].

**Figure 3:**
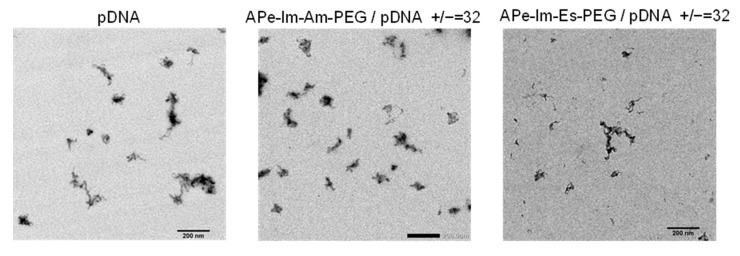
Representative TEM images of the APe-Im-Am-PEG/pDNA or APe-Im-Es-PEG/pDNA MICs. The mixing charge (+/−) ratios of the APe-Im-Am-PEG or APe-Im-Es-PEG to pDNA are indicated. Each scale bar represents 200 nm.

As shown in Figure 3, TEM observations revealed that the morphology of the pDNA in the MIC seems to be less condensed [32] and not spherical [22], suggesting the flexible structure of both the APe-Im-Am-PEG/pDNA and APe-Im-Es-PEG MICs.

**Figure 4:**
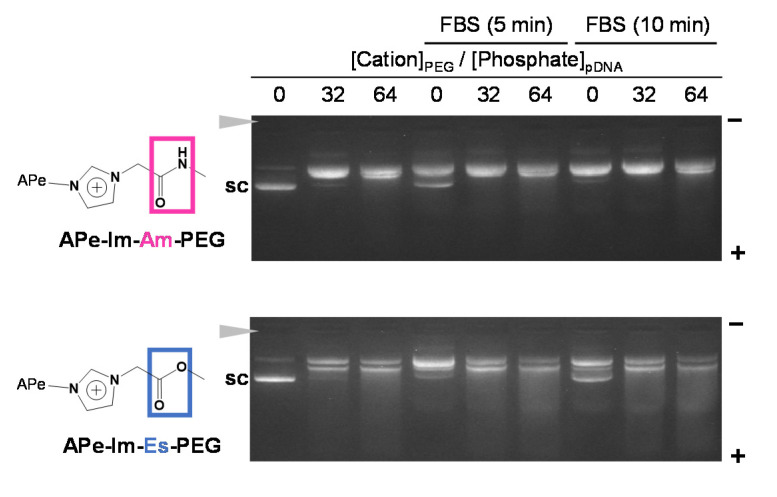
Stability assay of the APe-Im-Am-PEG/pDNA or APe-Im-Es-PEG/pDNA MICs ([*ω*-amide-pentylimidazolium (Cation)]_PEG_/[Phosphate]_pDNA_ (+/−) = 32 or 64) in serum protein by agarose gel electrophoresis. The MIC was incubated for 5 min or 10 min in the presence of 10% FBS, followed by loading to the gel. The solid arrowhead indicates the well where each sample was loaded. The migration band of super-coiled pDNA is indicated by “sc”.

As shown in Figure 4, to confirm the formation stability of the APe-Im-Am-PEG/pDNA and APe-Im-Es-PEG MICs in serum protein, in the viewpoint of pDNA delivery using the MIC by monovalent ionic interaction, we performed the agarose gel electrophoresis of the MICs in the presence of FBS. Although the digestion time with nuclease in FBS is shorter, as compared to many cases such as the use of deoxyribonuclease I (15 min) [37], the short digestion time is considered to be allowed from a viewpoint of human blood circulation time (approximately 1 min). The MIC bands were almost retained at the charge (+/−) ratios of 32 and 64 and no super-coiled pDNA bands (“sc” in Figure 4) were observed during the incubation of the APe-Im-Am-PEG/pDNA MIC with FBS even for 10 min. These results suggest the stable formation of the APe-Im-Am-PEG/pDNA MICs in the presence of FBS. During the incubation of the APe-Im-Es-PEG/pDNA MIC at the charge (+/−) ratios of 32 and 64, on the other hand, the MIC bands were slightly migrated into the plus pole and resultant slight bands appeared at the migration site of the super-coiled pDNA band (“sc” in Figure 4). These results suggest a little hydrolysis of the ester bond of the APe-Im-Es-PEG to form an unstable MIC.

### 3.3. Gene Expression of the APe-Im-Am-PEG/pDNA and APe-Im-Es-PEG MICs by Muscular Injection

**Figure 5:**
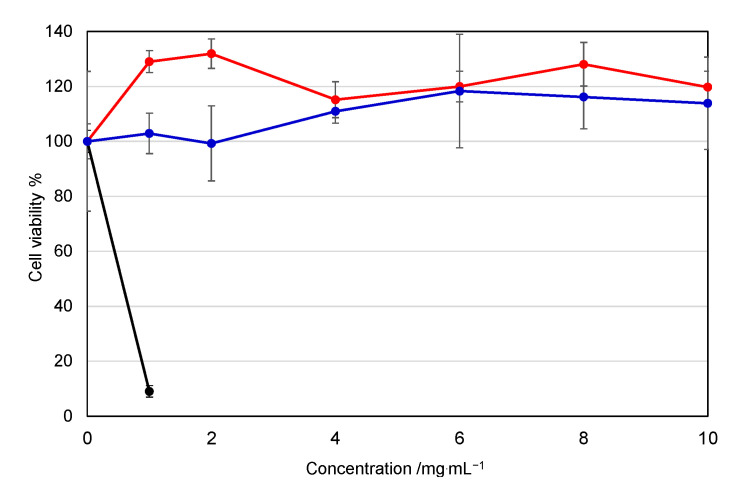
Effect of the APe-Im-Am-PEG or APe-Im-Es-PEG on the cell (HepG2) viability: APe-Im-Am-PEG (red), APe-Im-Es-PEG (blue), and bPEI (black: rapidly decreasing). Symbols and error bars represent the mean and standard deviation of the measurements made in triplicate wells.

The cytotoxicity of a mono-cationic PEG is an important factor for clinical application. Free mono-cationic PEGs exist solely when there is a release of pDNA from the MICs. Furthermore, the overall cytotoxicity of free mono-cationic PEGs is considered to be higher than that of the corresponding MICs. Therefore, we carried out the cytotoxicity assay of the free mono-cationic PEGs to give a worst-case interaction of the mono-cationic PEGs with cells rather than that of the MICs with pDNA. Figure 5 shows the effect of the APe-Im-Am-PEG or APe-Im-Es-PEG on the cell viability of human hepatoma HepG2 as a representative cell. The APe-Im-Am-PEG and APe-Im-Es-PEG maintained almost 100% cell viability, whereas branched poly(ethylenimine) (b-PEI) decreased cell viability. These results are consistent with our previous study that the *ω*-amide-pentylimidazolium group was a noncytotoxic cation [29,30]. Although toxicity in the liver, kidneys, etc., should be checked by hematological parameters [38], no damage of cellular membranes by cation species was emphasized because of the use of a local muscular injection, and not an intravenous injection, in this study.

**Figure 6:**
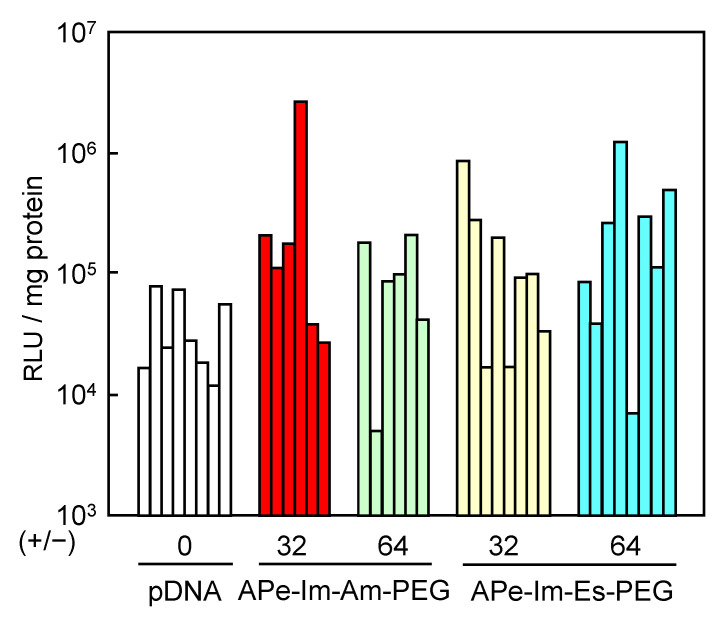
Luciferase gene expression by muscular injection of the APe-Im-Am-PEG/pDNA MIC or APe-Im-Es-PEG/pDNA MIC at [*ω*-amide-pentylimidazolium]_PEG_/[phosphate]_pDNA_ (+/−) ratios of 32 and 64. Individual gene expression was determined by relative light unit (RLU) normalized by protein concentration.

As a result of there being no apparent cytotoxicity, we finally examined the pDNA gene expression of the APe-Im-Am-PEG/pDNA and APe-Im-Es-PEG/pDNA MICs by muscular injection. After the injection, there was no change in the animal’s health and the appearance at the injection site, whereas turbidity was observed at the site by the use of in vivo transfection reagent PEI (commercially available in vivo-jetPEI™) (results not shown). These results suggest the biocompatibility based on the mono-cation property (minimum number of cation) of APe-Im-Am/Es-PEG as well as the PEGylation [25]. In the case of muscular injection, naked pDNAs are used for some clinical trials to mediate significant gene expression [1,2], resulting in first approval in Japan in 2019. As shown in Figure 6, the individual gene expression values mediated by both APe-Im-Am-PEG/pDNA MIC and APe-Im-Es-PEG/pDNA MIC seem to be generally higher than the expression values mediated by naked pDNA for clinical use. Therefore, we have carried out statistical analysis (Figure S3), resulting in the statistical significance (*p* < 0.1) of the average gene expression value of each MIC (+/− = 32 and 64) compared to the naked pDNA (+/− = 0). Especially, the highest gene expression value (2.7 × 10^6^ RLU/mg protein) was obtained by the APe-Im-Am-PEG/pDNA MIC at the charge (+/−) ratios of 32. The highest gene expression mediated by the APe-Im-Am-PEG/pDNA MIC at the charge (+/−) ratio of 32 was approximately 100 times higher than the average gene expression value mediated by naked pDNA (3.9 × 10^4^ RLU/mg protein). Furthermore, replicated data sets of the individual four experiments show a significant difference in gene expression mediated by the APe-Im-Am-PEG/pDNA MICs (*p* < 0.1) compared with the naked pDNA (Figure S4). Conversely, these data sets show no significant difference in those mediated by the APe-Im-Es-PEG/pDNA MICs (*p* > 0.1) compared with the naked pDNA (Figure S4). Moreover, the gene expression after one more week (total two weeks) also shows that the APe-Im-Am-PEG/pDNA MICs at the charge (+/−) ratio of 64 have especially better performance than the APe-Im-Es-PEG/pDNA MICs (Figure S5). These results suggest that the stable amide spacer between an *ω*-amide-pentylimidazolium group and a PEG chain is suitable for maximum gene expression in the skeletal muscles mediated by the pDNA MIC. This may be due to the higher stability of the APe-Im-Am-PEG/pDNA MIC, as compared to the APe-Im-Es-PEG/pDNA MIC, in the presence of FBS (Figure 4). In spite of maximum gene expression mediated by the APe-Im-Am-PEG/pDNA MIC based on the higher stability in the presence of FBS, the APe-Im-Es-PEG/pDNA MIC exhibited similar delivery and efficacy as that of the APe-Im-Am-PEG/pDNA MIC (Figure S3), suggesting the effect of the cleavage of PEG for the accessibility to target cells [30,31] and the production of a pH-sensitive carboxyl group on the side of APe-Im-Es-PEG [32] for endosomal escape [39,40]. Although the reason why an amide spacer was better is not exactly understood, we can conclude that the spacer structure between a terminal cation and a PEG chain is an important factor for the gene transfection activity of the corresponding pDNA MIC.

## 4. Conclusions

The pDNA MIC with the monocationic PEG with a stable amide (Am) spacer, as compared to a hydrolytic ester (Es) spacer, is considered to be suitable for the highest gene expression by muscular injection. In this study, the spacer structure between a terminal cation and a PEG chain is concluded to be an important factor for the gene transfection activity of the corresponding pDNA MIC, because the difference between the APe-Im-Am-PEG and APe-Im-Es-PEG was only a spacer structure between a terminal cation and a PEG chain. Consequently, the consideration of the spacer design for the monocationic PEG is essential to form the future pDNA MIC for the effective activity of gene transfection by muscular injection.

## Figures and Tables

**Table 1:** Particle size and zeta potential of APe-Im-Am-PEG and APe-Im-Es-PEG MICs.

	Cation)]_PEG_/[Phosphate]_pDNA_	Particle Size/nm	Zeta Potential/mV
APe-Im-Am-PEG/pDNA	32	48.2 ± 0.8	−6.04 ± 2.0
	64	65.1 ± 0.0	−10.8 ± 2.2
APe-Im-Am-PEG/pDNA	32	54.1 ± 11.7	−7.0 ± 1.1
	64	73.8 ± 11.3	−12.6 ± 3.0

## Data Availability

Data is contained within the article or supplementary materials.

## Supplementary Materials

The following are available online at https://www.mdpi.com/1999-4923/13/1/78/s1, Figure S1: ^1^H NMR spectra of APe-Im-Am-PEG and APe-Im-Es-PEG, Figure S2: Effect of incubation time on the hydrolysis of the APe-Im-Am-PEG and APe-Im-Es-PEG examined by gel-filtration chromatograms, Figure S3: Luciferase gene expression shown as mean and standard deviation, Figure S4: Replicated data sets of the individual animal experiments, Figure S5: Luciferase gene expression after two weeks by muscular injection.

## References

[1] Morishita R., Aoki M., Hashiya N., Makino H., Yamasaki K., Azuma J., Sawa Y., Matsuda H., Kaneda Y., Ogihara T. (2004). Safety evaluation of clinical gene therapy using hepatocyte growth factor to treat peripheral arterial disease. Hypertension.

[2] Shigematsu H., Yasuda K., Iwai T., Sasajima T., Ishimaru S., Ohashi Y., Yamaguchi T., Ogihara T., Morishita R. (2010). Randomized, double-blind, placebo-controlled clinical trial of hepatocyte growth factor plasmid for critical limb ischemia. Gene Ther..

[3] Raftery R.M., Mencía Castaño I., Chen G., Cavanagh B., Quinn B., Curtin C.M., Cryan S.A., O’Brien F.J. (2017). Translating the role of osteogenic-angiogenic coupling in bone formation: Highly efficient chitosan-pDNA activated scaffolds can accelerate bone regeneration in critical-sized bone defects. Biomaterials.

[4] Dos Santos Rodrigues B., Oue H., Banerjee A., Kanekiyo T., Singh J. (2018). Dual functionalized liposome-mediated gene delivery across triple co-culture blood brain barrier model and specific in vivo neuronal transfection. J. Control. Release.

[5] Phillips H.R., Tolstyka Z.P., Hall B.C., Hexum J.K., Hackett P.B., Reineke T.M. (2019). Glycopolycation-DNA Polyplex Formulation N/P Ratio Affects Stability, Hemocompatibility, and In Vivo Biodistribution. Biomacromolecules.

[6] Lin F., Shen X., Kichaev G., Mendoza J.M., Yang M., Armendi P., Yan J., Kobinger G.P., Bello A., Khan A.S. (2012). Optimization of electroporation-enhanced intradermal delivery of DNA vaccine using a minimally invasive surface device. Hum. Gene Ther. Methods.

[7] Duperret E.K., Trautz A., Stoltz R., Patel A., Wise M.C., Perales-Puchalt A., Smith T., Broderick K.E., Masteller E., Kim J.J. (2018). Synthetic DNA-Encoded Monoclonal Antibody Delivery of Anti-CTLA-4 Antibodies Induces Tumor Shrinkage In Vivo. Cancer Res..

[8] Kwak S.Y., Han H.D., Ahn H.J. (2019). A T7 autogene-based hybrid mRNA/DNA system for long-term shRNA expression in cytoplasm without inefficient nuclear entry. Sci. Rep..

[9] Thomas M., Klibanov A.M. (2003). Conjugation to gold nanoparticles enhances polyethylenimine’s transfer of plasmid DNA into mammalian cells. Proc. Natl. Acad. Sci. USA.

[10] Talsma S.S., Babensee J.E., Murthy N., Williams I.R. (2006). Development and in vitro validation of a targeted delivery vehicle for DNA vaccines. J. Control. Release.

[11] Asayama S., Sekine T., Kawakami H., Nagaoka S. (2007). Design of aminated poly(1-vinylimidazole) for a new pH-sensitive polycation to enhance cell-specific gene delivery. Bioconjugate Chem..

[12] Asayama S., Sudo M., Nagaoka S., Kawakami H. (2008). Carboxymethyl poly(L-histidine) as a new pH-sensitive polypeptide to enhance polyplex gene delivery. Mol. Pharm..

[13] Asayama S., Hakamatani T., Kawakami H. (2010). Synthesis and characterization of alkylated poly(1-vinylimidazole) to control the stability of its DNA polyion complexes for gene delivery. Bioconjugate Chem..

[14] Asayama S., Nishinohara S., Kawakami H. (2011). Zinc-chelated imidazole groups for DNA polyion complex formation. Metallomics.

[15] Asayama S., Nishinohara S., Kawakami H. (2011). Zinc-chelated poly(1-vinylimidazole) and a carbohydrate ligand polycation form DNA ternary complexes for gene delivery. Bioconjugate Chem..

[16] Asayama S., Matsuda K., Negishi Y., Kawakami H. (2014). Intracellular co-delivery of zinc ions and plasmid DNA for enhancing gene transfection activity. Metallomics.

[17] Asayama S., Kumagai T., Kawakami H. (2015). Screening for methylated poly(L-histidine) with various dimethylimidazolium/methylimidazole/imidazole contents as DNA carrier. Pharmaceutics.

[18] Liu C., Zhang L., Liu H., Cheng K. (2017). Delivery strategies of the CRISPR-Cas9 gene-editing system for therapeutic applications. J. Control. Release.

[19] Ji Y., Liu X., Huang M., Jiang J., Liao Y.P., Liu Q., Chang C.H., Liao H., Lu J., Wang X. (2019). Development of self-assembled multi-arm polyrotaxanes nanocarriers for systemic plasmid delivery in vivo. Biomaterials.

[20] Asayama S. (2020). Molecular design of polymer-based carriers for plasmid DNA delivery *in vitro* and *in vivo*. Chem. Lett..

[21] Wang J., Lee I.L., Lim W.S., Chia S.M., Yu H., Leong K.W., Mao H.Q. (2004). Evaluation of collagen and methylated collagen as gene carriers. Int. J. Pharm..

[22] Kobayashi Y., Nirasawa K., Negishi Y., Asayama S. (2020). Noncorrelative relation between *in vitro* and *in vivo* for plasmid DNA transfection by succinylated polyethylenimine muscular injection. J. Biomater. Sci. Polym. Ed..

[23] Durymanov M.O., Slastnikova T.A., Kuzmich A.I., Khramtsov Y.V., Ulasov A.V., Rosenkranz A.A., Egorov S.Y., Sverdlov E.D., Sobolev A.S. (2013). Microdistribution of MC1R-targeted polyplexes in murine melanoma tumor tissue. Biomaterials.

[24] Durymanov M.O., Yarutkin A.V., Bagrov D.V., Klinov D.V., Kedrov A.V., Chemeris N.K., Rosenkranz A.A., Sobolev A.S. (2016). Application of vasoactive and matrix-modifying drugs can improve polyplex delivery to tumors upon intravenous administration. J. Control. Release.

[25] Chen Q., Osada K., Ishii T., Oba M., Uchida S., Tockary T.A., Endo T., Ge Z., Kinoh H., Kano M.R. (2012). Homo-catiomer integration into PEGylated polyplex micelle from block-catiomer for systemic anti-angiogenic gene therapy for fibrotic pancreatic tumors. Biomaterials.

[26] Gao H., Liu J., Yang C., Cheng T., Chu L., Xu H., Meng A., Fan S., Shi L., Liu J. (2013). The impact of PEGylation patterns on the in vivo biodistribution of mixed shell micelles. Int. J. Nanomed..

[27] Nance E., Zhang C., Shih T.Y., Xu Q., Schuster B.S., Hanes J. (2014). Brain-penetrating nanoparticles improve paclitaxel efficacy in malignant glioma following local administration. ACS Nano.

[28] Asayama S., Nohara A., Negishi Y., Kawakami H. (2014). Alkylimidazolium end-modified poly(ethylene glycol) to form the mono-ion complex with plasmid DNA for in vivo gene delivery. Biomacromolecules.

[29] Asayama S., Nohara A., Negishi Y., Kawakami H. (2015). Plasmid DNA mono-ion complex stabilized by hydrogen bond for in vivo diffusive gene delivery. Biomacromolecules.

[30] Mishra S., Webster P., Davis M.E. (2004). PEGylation significantly affects cellular uptake and intracellular trafficking of non-viral gene delivery particles. Eur. J. Cell Biol..

[31] Hatakeyama H., Akita H., Harashima H. (2011). A multifunctional envelope type nano device (MEND) for gene delivery to tumors based on the EPR effect: A strategy for overcoming the PEG dilemma. Adv. Drug Deliv. Rev..

[32] Kobayashi Y., Taneichi S., Kawakami H., Negishi Y., Asayama S. (2019). Plasmid DNA Mono-Ion Complex for in Vivo Sustainable Gene Expression. ACS Omega.

[33] Unsworth J.M., Rose F.R.A.J., Wright E., Scotchford C.A., Shakesheff K.M. (2003). Seeding cells into needled felt scaffolds for tissue engineering applications. J. Biomed. Mater. Res..

[34] Negishi Y., Matsuo K., Endo-Takahashi Y., Suzuki K., Matsuki Y., Takagi N., Suzuki R., Maruyama K., Aramaki Y. (2011). Delivery of an angiogenic gene into ischemic muscle by novel bubble liposomes followed by ultrasound exposure. Pharm. Res..

[35] Wakebayashi D., Nishiyama N., Yamasaki Y., Itaka K., Kanayama N., Harada A., Nagasaki Y., Kataoka K. (2004). Lactose-conjugated polyion complex micelles incorporating plasmid DNA as a targetable gene vector system: Their preparation and gene transfecting efficiency against cultured HepG2 cells. J. Control. Release.

[36] Itaka K., Yamauchi K., Harada A., Nakamura K., Kawaguchi H., Kataoka K. (2003). Polyion complex micelles from plasmid DNA and poly(ethylene glycol)-poly(L-lysine) block copolymer as serum-tolerable polyplex system: Physicochemical properties of micelles relevant to gene transfection efficiency. Biomaterials.

[37] Pan S., Cao D., Yi W., Huang H., Feng M. (2013). A biodegradable and serum-resistant gene delivery carrier composed of polyamidoamine-poly *N*,*N*′-di-(2-aminoethyl) aminoethyl glutamine copolymer. Colloids Surf. B Biointerfaces.

[38] Hashiba A., Toyooka M., Sato Y., Maeki M., Tokeshi M., Harashima H. (2020). The use of design of experiments with multiple response to determine optimal formulations for *in vivo* hepatic mRNA delivery. J. Control. Release.

[39] Yuba E., Kojima C., Sakaguchi N., Harada A., Koiwai K., Kono K. (2008). Gene delivery to dendritic cells mediated by complexes of lipoplexes and pH-sensitive fusogenic polymer-modified liposomes. J. Control. Release.

[40] Wu Y., Wu J., Cao J., Zhang Y., Xu Z., Qin X., Wang W., Yuan Z. (2017). Facile fabrication of poly(acrylic acid) coated chitosan nanoparticles with improved stability in biological environments. Eur. J. Pharm. Biopharm..

