# Supplementary material for: Structure-Activity Relationship of Mono-Ion Complexes for Plasmid DNA Delivery by Muscular Injection
Source: Pharmaceutics. 2021 Jan 8;13(1):78. doi: 10.3390/pharmaceutics13010078 (PMC7828051; doi:10.3390/pharmaceutics13010078)
Supplement: Supplementary file 1 [file pharmaceutics-13-00078-s001.pdf]

pharmaceutics

Supplementary Materials: Structure-Activity Relationship of Mono-Ion Complexes for Plasmid DNA Delivery by Muscular Injection

Amika Mori, Yuki Kobayashi, Kei Nirasawa, Yoichi Negishi and Shoichiro Asayama*

Table of Contents

1. ^{1}H NMR spectra of APe-Im-Am-PEG and APe-Im-Es-PEG (Figure S1)
2. Effect of incubation time on the hydrolysis of APe-Im-Am-PEG and APe-Im-Es-PEG examined by gel-filtration chromatograms (Figure S2)
3. Luciferase gene expression shown as mean and standard deviation (Figure S3)
4. Replicated data sets of the individual animal experiments (Figure S4)
5. Luciferase gene expression after two weeks by muscular injection (Figure S5)

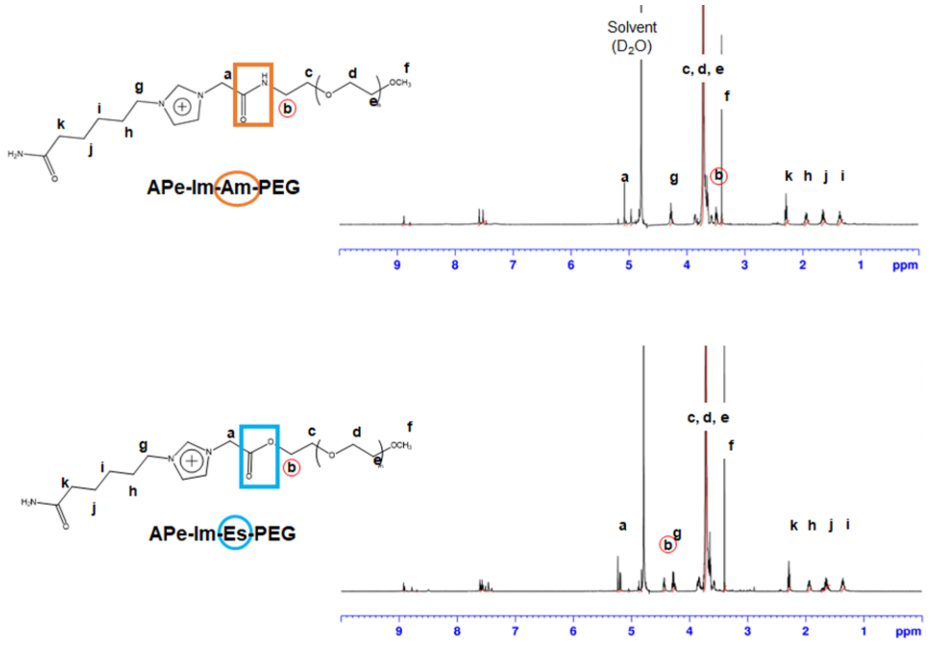

Figure S1. ^{1}H NMR spectra of APe-Im-Am-PEG and APe-Im-Es-PEG.

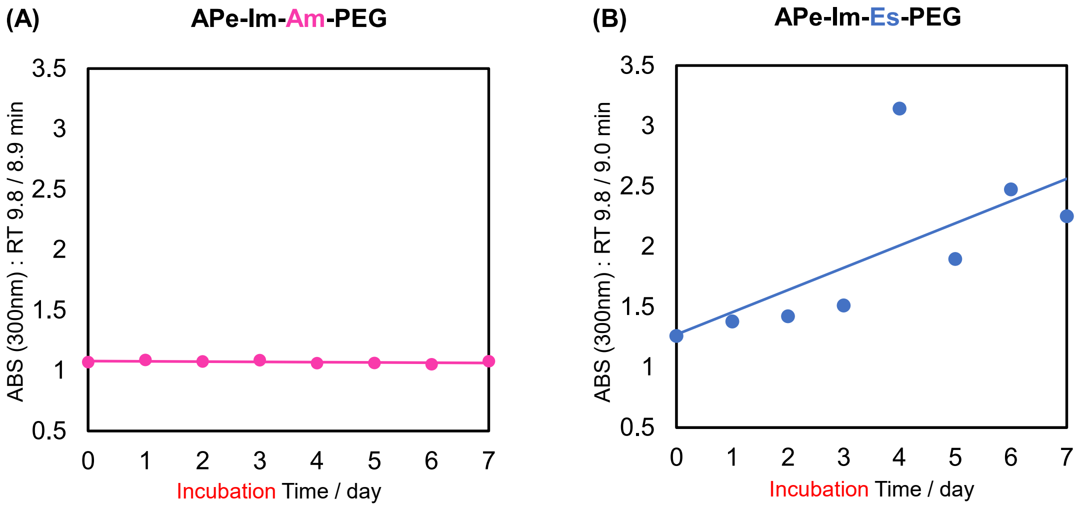

Figure S2. Effect of incubation time on the hydrolysis of APe-Im-Am-PEG (A) and APe-Im-Es-PEG (B) examined by gel-filtration chromatograms. Detection: absorbance (ABS) at 300 nm as the ratio of each retention time (RT).

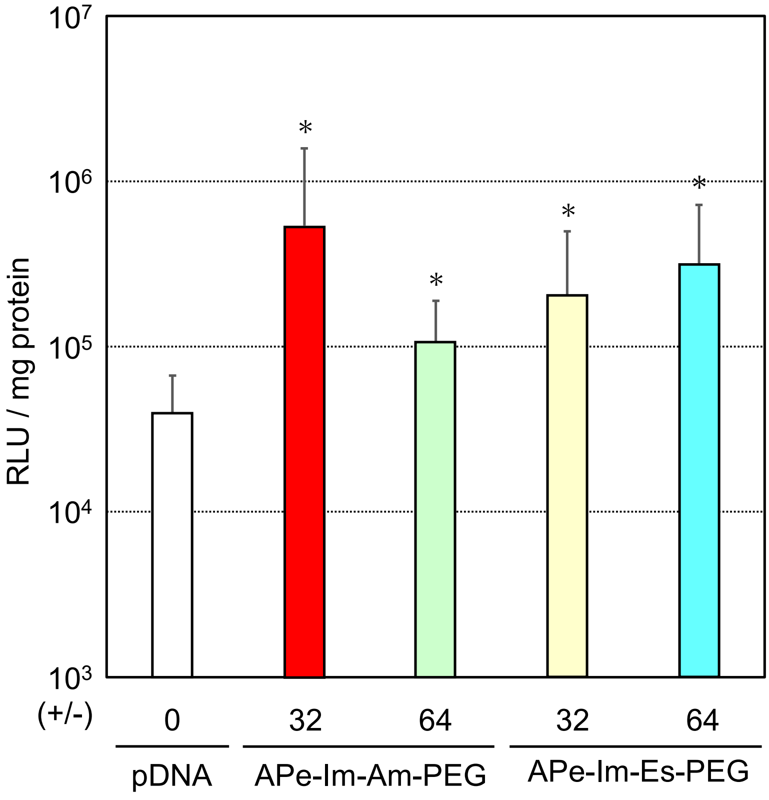

Figure S3. Luciferase gene expression by muscular injection of the APe-Im-Am-PEG/pDNA MIC or APe-Im-Es-PEG/pDNA MIC at [ω-amide-pentylimidazolium]$_{PEG}$/[phosphate]$_{pDNA}$ (+/-) ratios of 32 and 64. Data are shown as mean and standard deviation based on individual data in Figure 6. The * indicates statistical significance ($p < 0.1$) compared with the naked pDNA.

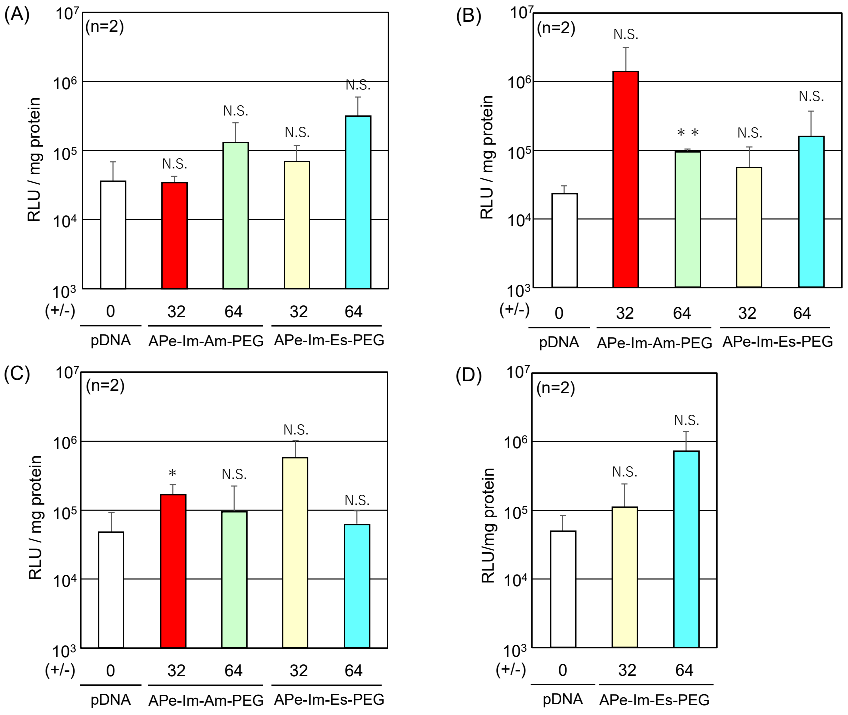

Figure S4. Replicated data sets (A-D) of the individual animal experiments (n = 2) base on an average of all data in Figure S3. Luciferase gene expression by muscular injection of the APe-Im-Am-PEG/pDNA MIC or APe-Im-Es-PEG/pDNA MIC at [ω-amide-pentylimidazolium]$_{PEG}$/[phosphate]$_{pDNA}$ (+/-) ratios of 32 and 64. Data are shown as mean and standard deviation based on individual data in Figure 6. The statistical significance (*p < 0.1, **p < 0.01) compared with the naked pDNA is indicated; N.S., not significant statistical difference (p > 0.1).

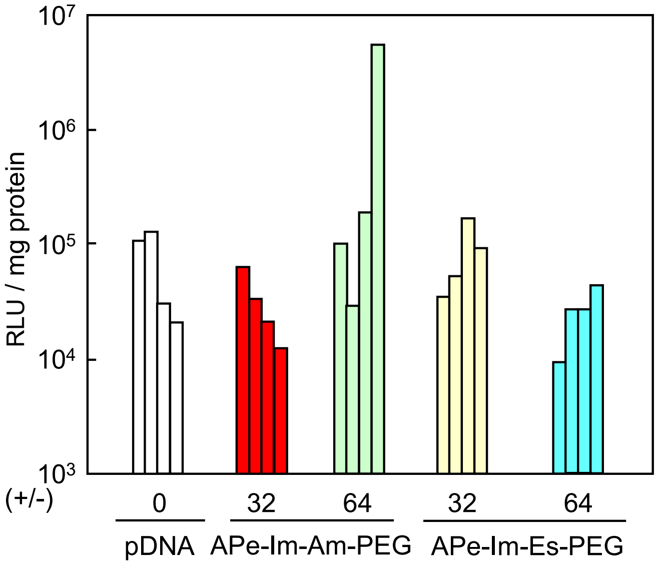

Figure S5. Luciferase gene expression after two weeks by muscular injection of the APe-Im-Am-PEG/pDNA MIC or APe-Im-Es-PEG/pDNA MIC at [ω-amide-pentylimidazolium]$_{PEG}$/[phosphate]$_{pDNA}$ (+/-) ratios of 32 and 64. Individual gene expression was determined relative light unit (RLU) normalized by the protein concentration.
